# Neutrophil-to-albumin ratio as a predictor of sepsis-associated encephalopathy in patients with rheumatoid arthritis: an exploratory machine learning study

**DOI:** 10.3389/fneur.2026.1884282

**Published:** 2026-07-23

**Authors:** Tong Xin, Che Wang, Fan Gong, Yanxia Zhao, Jingjing He, Xiaoping Wang, Daqing Nie, Guorong Jin, Aijing Liu

**Affiliations:** 1 Department of Rheumatology and Immunology, The Second Hospital of Hebei Medical University, Shijiazhuang, Hebei, China; 2 Hebei International Joint Research Center on Rheumatic Diseases, Shijiazhuang, Hebei, China; 3 Department of Medical and Pharmaceutical Informatics, Hebei Medical University, Shijiazhuang, Hebei, China; 4 Department of Rheumatology, The Affiliated Hospital to Changchun University of Chinese Medicine, Changchun, Jilin, China; 5 Department of Spine and Traumatic Orthopedics, Beibei Hospital of Chongqing Medical University, Chongqing, China; 6 Hebei Key Laboratory of Laboratory Medicine, Shijiazhuang, Hebei, China

**Keywords:** machine learning, neutrophil-to-albumin ratio, rheumatoid arthritis, sepsis-associated encephalopathy, XGBoost

## Abstract

**Background and purpose:**

Patients with rheumatoid arthritis (RA) face an elevated risk of sepsis and its associated neurological complications, most notably sepsis-associated encephalopathy (SAE). Nonetheless, early identification of SAE in this population remains a substantial clinical challenge. To address this gap, we aimed to develop and validate a predictive model that incorporates the neutrophil-to-albumin ratio (NAR) to estimate SAE risk in RA patients with sepsis.

**Methods:**

This retrospective multicenter cohort study included a derivation cohort of 89 patients with RA and sepsis from two centers and an independent external validation cohort of 37 patients from a third center. Patients in the derivation cohort were classified into SAE and non-SAE groups. Three machine learning algorithms (LASSO, random forest, and XGBoost) were applied for feature selection, and the optimal model was selected based on area under the receiver operating characteristic curve (AUC). Model performance was evaluated using bootstrap resampling, calibration curves, and decision curve analysis. The final XGBoost model was subsequently evaluated in the external validation cohort. A web-based dynamic prediction tool was developed for clinical application. Kaplan–Meier analysis and Cox regression were performed to evaluate 28-day survival.

**Results:**

SAE occurred in 23.6% of patients and was associated with significantly higher 28-day mortality (61.9% vs. 33.8%, *p* = 0.04). Six consensus predictors (SOFA score, procalcitonin, platelet count, length of stay, NAR, and age) were identified. The XGBoost model achieved a bootstrap-corrected AUC of 0.859 (95% CI: 0.751–0.946) in the derivation cohort. In the independent external validation cohort, the final XGBoost model achieved an AUC of 0.849 (95% CI: 0.674–1.000). SHAP analysis demonstrated that higher SOFA, procalcitonin, length of stay, age, and NAR values increased SAE risk, whereas higher platelet count was protective. Kaplan–Meier analysis showed significantly lower 28-day survival in the high NAR group (*p* = 0.023), and elevated NAR was associated with increased mortality risk (HR = 2.178, 95% CI: 1.095–4.332).

**Conclusion:**

The NAR-integrated XGBoost model provides a robust and interpretable tool for early SAE prediction in RA patients with sepsis, showing potential clinical utility for bedside risk stratification.

## 1 Introduction

Rheumatoid arthritis (RA) is a chronic systemic autoimmune disease characterized by persistent immune dysregulation and sustained inflammatory activity (1, 2). Patients with RA are at substantially increased risk of severe infections and sepsis due to disease-related immune dysfunction and prolonged exposure to immunosuppressive therapies, including glucocorticoids and biologic disease-modifying antirheumatic drugs (DMARDs) (3, 4). Importantly, accumulating evidence suggests that RA patients are not only at increased risk of infection, but also more likely to experience severe or complicated disease courses once infected (4). This risk is further amplified by advanced age, multimorbidity, and persistent systemic inflammation, all of which contribute to impaired physiological reserve. Sepsis represents a major and life-threatening complication in RA patients. Among its complications, sepsis-associated encephalopathy (SAE) is associated with increased mortality and poor clinical outcomes (5). Given the combination of immune dysregulation, systemic inflammation, and vulnerable host status, RA patients may represent a high-risk subgroup for adverse sepsis-related outcomes. However, SAE risk stratification in this population remains understudied.

Recent observational and Mendelian randomization studies have suggested a potential causal association between autoimmune diseases, particularly RA, and both the occurrence and short-term mortality of sepsis (6). However, these studies have primarily focused on systemic infectious outcomes. In contrast, neurological complications of sepsis in RA patients remain largely unexplored. To date, evidence regarding SAE risk stratification and prognostic features in RA patients is still lacking.

Despite its clinical significance, early identification of SAE remains challenging due to its nonspecific clinical manifestations and the absence of reliable biomarkers (7). Conventional severity scores, such as the Sequential Organ Failure Assessment (SOFA) score, are designed for general sepsis populations and may not fully capture the unique inflammatory and immunological characteristics of RA patients (8). Therefore, there is an unmet need for simple and readily available biomarkers to improve early risk stratification. The neutrophil-to-albumin ratio (NAR), which integrates systemic inflammation and nutritional status, has recently emerged as a promising prognostic indicator in critically ill populations (9–11). Importantly, emerging evidence has suggested that the NAR is associated with adverse outcomes in cerebrovascular diseases. In patients with acute ischemic stroke, elevated NAR has been independently associated with poor functional outcomes (9), while in intracerebral hemorrhage, higher NAR levels have been linked to increased short-term mortality in multicenter cohort studies, supporting its potential role as a readily available prognostic biomarker in neurological and critically ill populations (10). However, its predictive value for SAE and short-term outcomes in patients with RA and sepsis remains unclear.

In this study, we aimed to develop a predictive model for SAE using a two-center derivation cohort and externally validate it in an independent cohort from a third center in patients with RA and sepsis. The model was constructed using NAR combined with routinely available clinical and laboratory parameters to facilitate early risk stratification and improve prognostic assessment in this high-risk population.

## 2 Methods

### 2.1 Study design and patients

This retrospective multicenter cohort study enrolled consecutive patients with RA complicated by sepsis who were admitted to the intensive care units (ICUs) of the Second Hospital of Hebei Medical University and the Beibei Hospital Affiliated to Chongqing Medical University between September 2020 and September 2025. Patients were categorized into SAE and non-SAE groups according to whether SAE occurred during hospitalization. Patients from these two centers formed the derivation cohort used for model development. An independent external validation cohort was subsequently obtained from the Affiliated Hospital of Changchun University of Chinese Medicine. Because the external cohort was used solely for validation of the final prediction model, data collection was limited to the six predictor variables required by the final extreme gradient boosting (XGBoost) model (age, NAR, SOFA score, procalcitonin [PCT], platelet count [PLT], and length of stay [LOS]).

Inclusion criteria were as follows: (1) age ≥18 years; (2) diagnosis of RA according to the 2010 American College of Rheumatology/European League Against Rheumatism (ACR/EULAR) classification criteria; (3) diagnosis of sepsis based on the Sepsis-3 definition (12); and (4) complete laboratory data, including Neutrophil count (NEU) and serum albumin (ALB) levels obtained within 24 h after sepsis onset. SAE was identified by documented cognitive or neuropsychiatric abnormalities, Glasgow Coma Scale (GCS) score <15, or delirium manifestations during sepsis after exclusion of other neurological causes. A multidisciplinary consensus adjudication process involving senior rheumatologists and neurocritical care specialists was implemented to differentiate SAE from other Central nervous system (CNS) disorders, including RA-related neurological involvement and autoimmune encephalitis. Cases with uncertain neurological classification, particularly those requiring differentiation between SAE and RA-related neurological involvement, were jointly reviewed by senior rheumatologists and neurocritical care specialists, and the final diagnosis was established through consensus after exclusion of alternative neurological diagnoses (13). Alternative neurological conditions were excluded based on clinical assessment and, when clinically indicated, cerebrospinal fluid analysis. Patients with indeterminate neurological status or insufficient diagnostic information were excluded to ensure diagnostic specificity. Additional exclusion criteria encompassed alternative causes of acute encephalopathy, broadly categorized as central nervous system autoimmune disorders (e.g., autoimmune encephalitis and CNS vasculitis), hyperinflammatory syndromes (e.g., hemophagocytic lymphohistiocytosis), structural or infectious brain diseases (e.g., traumatic brain injury and primary intracranial infection), metabolic encephalopathy (including hypoglycaemia, diabetic ketoacidosis, hepatic encephalopathy, pulmonary encephalopathy, and uraemic encephalopathy), toxic encephalopathy, and drug- or sedative-related cognitive impairment. Ultimately, 89 patients were included in the final analysis.

### 2.2 Data preprocessing

Missing data patterns were first assessed using Little’s missing completely at random (MCAR) test, which rejected the MCAR assumption (*p* < 0.05), indicating that the missingness did not meet the MCAR assumption. Given the relatively low proportion of missing values and the observed missingness patterns and clinical context, subsequent analyses were conducted under the assumption that the data were missing at random (MAR). Accordingly, multiple imputation was performed using two nonparametric approaches: k-nearest neighbors (kNN) imputation and random forest-based imputation. Post-imputation diagnostics showed that the distributions of the imputed variables were well preserved compared with the original data, and no obvious systematic bias was observed (14, 15). To reduce the influence of extreme values on subsequent analyses, all continuous variables were Winsorized at the 5th and 95th percentiles after imputation (14, 16).

### 2.3 Outcome

The primary outcome was SAE occurrence during hospitalization. SAE status was determined using the predefined diagnostic criteria and multidisciplinary consensus adjudication described above.

### 2.4 Candidate variables

Candidate variables included demographic characteristics, inflammatory markers, hematologic and biochemical parameters, and disease severity scores collected within 24 h after sepsis onset. Variables with >20% missing values were excluded to limit imputation-related bias.

The variables retained for analysis included gender, age, LOS history of diabetes mellitus, history of hypertension, coronary artery disease (CAD), RA duration, steroid use, white blood cell count (WBC), C-reactive protein (CRP), NEU lymphocyte count (LYM), monocyte count (MONO), red blood cell count (RBC), hemoglobin (Hb), PLT ALB globulin (GLB), PCT D-dimer, rheumatoid factor (RF), creatine kinase (CK), lactate dehydrogenase (LDH), anti-cyclic citrullinated peptide antibody (anti-CCP), Disease Activity Score in 28 joints using CRP (DAS28-CRP) (17), SOFA score, Acute Physiology and Chronic Health Evaluation II (APACHE II) score, 28-day mortality, as well as inflammation-related composite indices, including NAR, neutrophil-to-lymphocyte ratio (NLR), platelet-to-lymphocyte ratio (PLR), and systemic immune-inflammation index (SII).

### 2.5 Definition of clinical variables and calculation of derived indices

We standardized all index calculations across multiple clinical centers using routine laboratory parameters. The NAR was defined as: $\mathrm{NAR}=\frac{\mathrm{NEU}}{\mathrm{ALB}}$ , where NEU and ALB denote neutrophil count (10^9^/L) and serum albumin level (g/L), respectively. To improve interpretability in regression analyses, NAR values were multiplied by 10 prior to modeling. The NLR was calculated as: $\mathrm{NLR}=\frac{\mathrm{NEU}}{\mathrm{LYM}}$ , where LYM denotes lymphocyte count (10^9^/L). The PLR was defined as: $\mathrm{PLR}=\frac{\mathrm{PLT}}{\mathrm{Lym}}$ , where PLT represents platelet count (10^9^/L). The SII was calculated as: $\mathrm{SII}=\frac{\mathrm{PLT}\times \mathrm{NEU}}{\mathrm{Lym}}$ . Disease activity of RA was assessed using DAS28-CRP, calculated as: $\begin{array}{l} \mathrm{DAS}28-\mathrm{CRP}=0.56\times \sqrt{\mathrm{TJC}28}+0.28\times \sqrt{\mathrm{SJC}28}+0.014\times \mathrm{GH}\\ +0.36\times \ln(\mathrm{CRP}+1)+0.96\; \end{array}$ , where TJC28 and SJC28 represent tender and swollen 28-joint counts, respectively, CRP refers to the C-reactive protein level (mg/L), and GH denotes the patient’s global health assessment on a 0–100 mm visual analogue scale, with 0 indicating the best and 100 indicating the worst health status (17).

### 2.6 Variable selection and prediction model construction

Feature selection was performed using three machine learning-based algorithms, including least absolute shrinkage and selection operator (LASSO) regression (18), random forest (RF-ML) (19), and XGBoost (20), representing complementary modeling strategies including linear regularization, bagging-based nonlinear learning, and boosting-based ensemble learning, respectively. This combination was chosen to improve the robustness of feature selection by capturing predictors under different statistical assumptions and reducing potential model-specific bias. For each algorithm, the top 10 variables were selected according to feature importance rankings, or according to the absolute value of regression coefficients for LASSO. Variables common to all three top-10 feature sets were retained as consensus predictors for subsequent model construction.

Predictive models were subsequently constructed based on the selected features, and model performance was evaluated using the area under the receiver operating characteristic curve (AUC), with the model achieving the highest AUC selected as the final model for deployment. Considering the limited sample size, resampling-based internal validation methods were adopted instead of split-sample validation to maximize data utilization and avoid loss of statistical efficiency. Specifically, repeated 5-fold cross-validation with three repeats was used for model training and performance estimation, and bootstrap resampling with 1,000 iterations was further applied to assess model stability and optimism in predictive performance (21). In addition, external validation was further incorporated to evaluate model robustness and generalizability (22).

Incremental predictive performance of NAR was further evaluated by comparing the baseline model and the NAR-enhanced model using changes in AUC, Net Reclassification Improvement (NRI), and Integrated Discrimination Improvement (IDI). A complete-case sensitivity analysis was conducted by excluding patients with missing values in any of the variables included in the final machine learning model, and receiver operating characteristic (ROC) analysis was repeated to assess whether model discrimination was materially affected by the imputation procedure. For the incremental comparison, both the baseline and NAR-enhanced models were evaluated using repeated five-fold cross-validation with three repeats. Their ROC curves were compared using DeLong’s test, and the corresponding 95% confidence intervals (CIs) for the AUCs were estimated using the DeLong method.

A conventional multivariable logistic regression model was constructed using the same predictors as the final machine learning model, and comparative analyses between the machine learning and logistic regression models were subsequently performed. Model calibration was assessed using calibration curves to evaluate agreement between predicted probabilities and observed outcomes. Clinical utility across prediction models was evaluated using decision curve analysis (DCA) by comparing the net benefit across a range of threshold probabilities. Model interpretability was assessed using Shapley additive explanations (SHAP).

### 2.7 Web-based prediction tool development

A web-based dynamic prediction tool was developed by integrating the final machine learning model into an interactive application to facilitate clinical use. The model was implemented to allow real-time calculation of the probability of RA-SAE based on individual patient clinical and laboratory variables. The application enables users to input selected predictors and immediately obtain individualized risk estimates, thereby translating the predictive model into a clinically applicable decision-support tool. This tool was designed to enhance clinical interpretability and promote the practical application of the model in bedside risk stratification.

### 2.8 Correlation and survival analyses

Pairwise correlation analyses were performed using Spearman rank correlation coefficients across the SAE group, non-SAE group, and the overall cohort. Correlation coefficients were visualized using circular heatmap representations. For survival analysis, the clinical endpoint was 28-day mortality. Survival time was defined as the interval from hospital admission to death or censoring at 28 days. Baseline NAR was dichotomized based on the cohort median. Cumulative survival probabilities were estimated using the Kaplan–Meier method and compared using the log-rank test. Hazard ratios (HRs) with 95% CIs were derived from univariate Cox proportional hazards regression models. Additional multivariable Cox proportional hazards regression analyses were performed. Model 1 was adjusted for age and sex, while Model 2 was further adjusted for SOFA score.

### 2.9 Statistical analysis

All statistical analyses were conducted using R software (version 4.4.2). Depending on data distribution, continuous variables were expressed as mean ± standard deviation or median with interquartile range. Group comparisons for continuous variables were performed using the independent-samples t test for normally distributed data, whereas the Mann–Whitney U test was applied when distributions were non-normal. Categorical variables were summarized as frequencies and percentages, and differences between groups were assessed using the chi-square test or Fisher’s exact test when appropriate.

### 2.10 Ethical considerations

All procedures involving human participants were conducted in accordance with the ethical standards of the institutional and/or national research committees and the principles of the 1964 Helsinki Declaration and its later amendments or comparable ethical standards. This retrospective multicenter study was approved by the Ethics Committees of the Second Hospital of Hebei Medical University (Approval No. 2025-R771), the Beibei Hospital Affiliated to Chongqing Medical University (Approval No. 2026-KE-LUNSHEN-016), and the Affiliated Hospital of Changchun University of Chinese Medicine (Changchun) (Approval No. CCZYFYKYLL2026-066). Owing to the retrospective nature of the study and the use of de-identified clinical data, the requirement for informed consent was waived by all ethics committees.

## 3 Result

### 3.1 Characteristics of patients enrolled in the study

**Figure 1:**
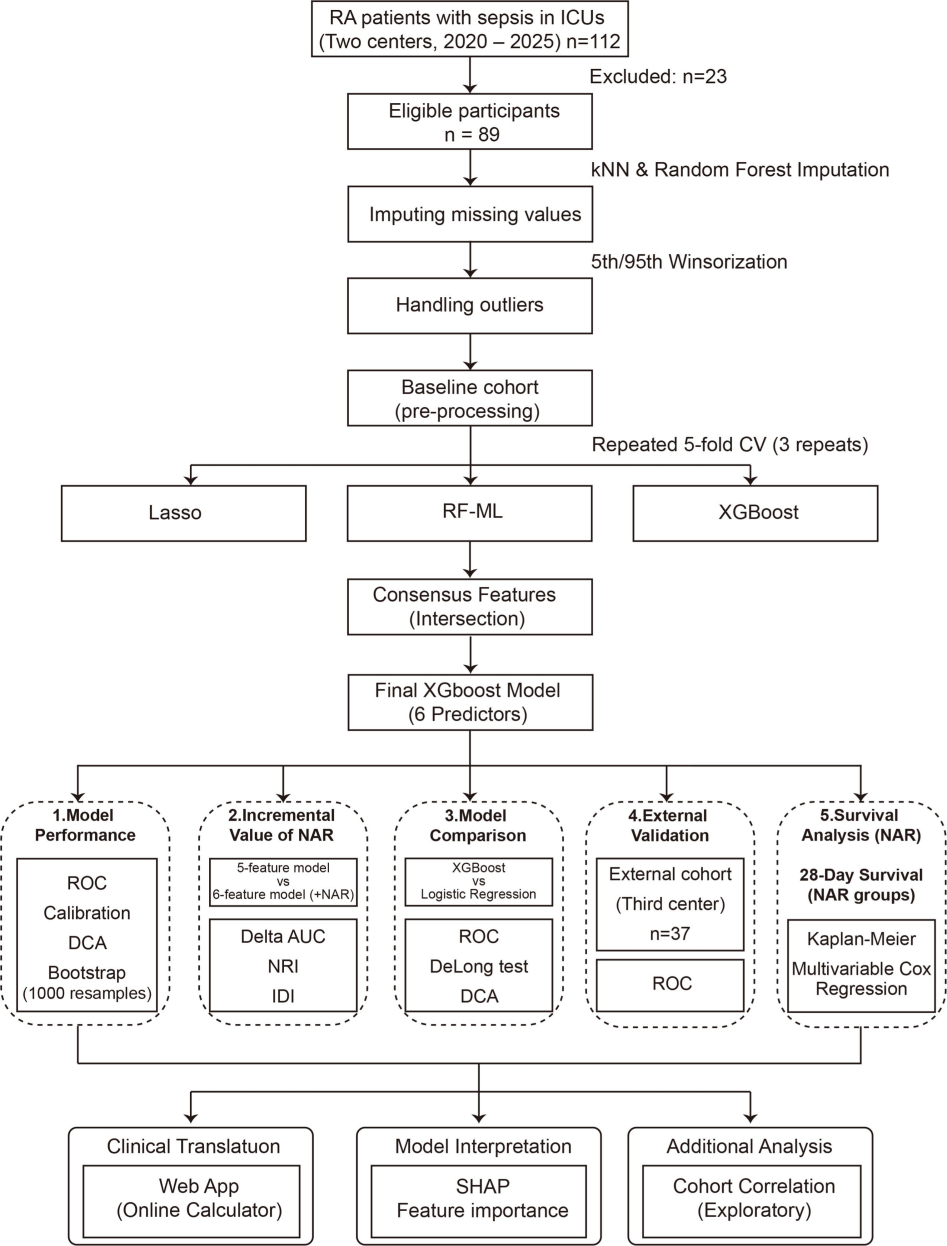
Flow diagram illustrating the model construction.

**Figure 2:**
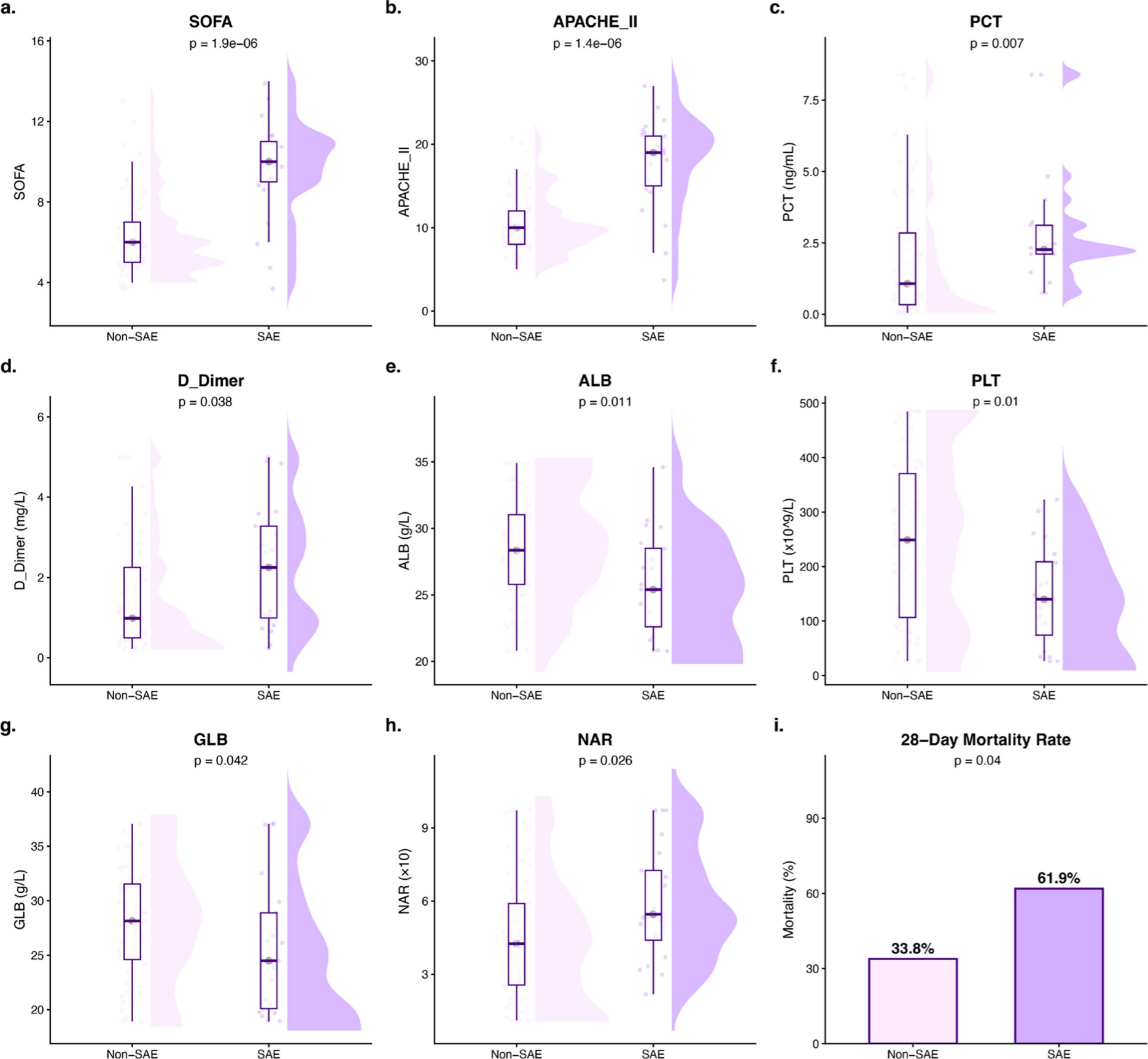
Comparison of clinical characteristics and inflammatory indices between Non-SAE and SAE groups. **(a–h)** Raincloud plots showing the distribution of SOFA score, APACHE II score, PCT, D-dimer, ALB, PLT, GLB, and NAR between the Non-SAE and SAE groups. The plots display data distributions using boxplots and kernel density estimation. **(i)** Comparison of 28-day mortality between the two groups. *p* < 0.05 was considered statistically significant.

The workflow of the study and model construction is illustrated in Figure 1, while details of data preprocessing and quality control are presented in Supplementary Figure S1 and described in Section 2.2. This study included 89 patients with RA complicated by sepsis, among whom 21 (23.6%) developed SAE. Compared with the non-SAE group, patients with SAE showed significantly lower PLT and ALB levels, but higher PCT D-dimer, SOFA, and APACHE II scores (all *p* < 0.05). Notably, NAR × 10 was significantly elevated in the SAE group compared with the non-SAE group (5.46 vs. 4.26, *p* = 0.026). In addition, the 28-day mortality rate was significantly higher in patients with SAE than in those without SAE (61.9% vs. 33.8%, *p* = 0.040). Detailed baseline characteristics are summarized in Table 1 and Figure 2.

**Table 1:** Characteristics of patients enrolled in the study.

Variables	Overall (*n* = 89)	Non-SAE (*n* = 68)	SAE (*n* = 21)	*p*-value
Demographics				
Gender, Female (%)	65 (73.0)	47 (69.1)	18 (85.7)	0.167
Age,years	65.80 (11.45)	65.12 (11.18)	68.00 (12.32)	0.316
LOS,days	11.00 [5.00, 17.00]	10.00 [5.00, 15.00]	12.00 [6.00, 22.00]	0.331
Clinical history				
Diabetes, No (%)	57 (64.0)	43 (63.2)	14 (66.7)	0.999
Hypertension, No (%)	48 (53.9)	36 (52.9)	12 (57.1)	0.806
CAD, No (%)	60 (67.4)	44 (64.7)	16 (76.2)	0.428
RA-Duration, years	11.00 [7.00, 20.00]	10.50 [6.50, 20.00]	15.00 [10.00, 20.00]	0.145
Steroid, use (%)	63 (70.8)	50 (73.5)	13 (61.9)	0.41
Laboratory tests				
WBC (×10^9^/L)	14.90 [10.21, 19.10]	13.79 [9.29, 19.12]	16.40 [13.80, 18.98]	0.088
CRP (mg/L)	143.78 [67.50, 212.00]	140.58 [64.12, 197.18]	173.10 [113.00, 252.60]	0.095
NEU (×10^9^/L)	12.94 [8.17, 17.11]	12.12 [7.88, 17.03]	13.92 [11.50, 17.20]	0.127
LYM (×10^9^/L)	0.94 [0.61, 1.47]	0.96 [0.61, 1.42]	0.92 [0.66, 1.59]	0.619
MONO (×10^9^/L)	0.55 [0.40, 0.83]	0.54 [0.39, 0.80]	0.65 [0.40, 0.92]	0.31
RBC (×10^12^/L)	3.51 [3.01, 3.88]	3.51 [3.04, 3.85]	3.33 [2.85, 3.90]	0.724
Hb (g/L)	102.00 [83.00, 115.00]	102.00 [87.50, 114.00]	103.00 [83.00, 118.00]	0.946
PLT (×10^9^/L)	207.00 [96.00, 316.00]	249.00 [106.50, 370.75]	140.00 [74.00, 209.00]	**0.01**
ALB (g/L)	27.70 [25.20, 30.50]	28.35 [25.78, 31.02]	25.40 [22.60, 28.50]	**0.011**
GLB (g/L)	27.70 [23.80, 31.20]	28.15 [24.60, 31.55]	24.50 [20.10, 28.90]	**0.042**
PCT (ng/mL)	1.56 [0.56, 3.01]	1.11 [0.34, 2.85]	2.27 [2.11, 3.12]	**0.007**
D-Dimer (mg/L)	1.05 [0.56, 2.63]	0.96 [0.50, 2.25]	2.25 [0.99, 3.28]	**0.038**
RF (IU/mL)	63.40 [25.10, 179.00]	50.85 [24.68, 173.75]	107.00 [43.00, 179.00]	0.373
CK (U/L)	51.00 [26.00, 105.00]	53.00 [25.00, 102.75]	45.60 [29.00, 220.00]	0.505
LDH (U/L)	290.80 [208.00, 484.00]	280.50 [213.25, 489.38]	332.50 [199.00, 459.00]	0.965
Anti-CCP (RU/mL)	238.00 [20.50, 883.25]	292.80 [19.07, 1021.84]	139.61 [85.50, 544.86]	0.969
Inflammatory indices and severity scores				
DAS28-CRP	5.48 (1.02)	5.48 (1.06)	5.48 (0.89)	0.983
NAR (×10)	4.67 [2.99, 6.43]	4.26 [2.56, 5.90]	5.46 [4.40, 7.26]	**0.026**
NLR	12.14 [7.90, 20.83]	11.62 [7.13, 20.70]	15.46 [9.56, 22.33]	0.236
PLR	152.87 [102.86, 257.58]	169.02 [98.77, 267.15]	138.33 [118.00, 257.14]	0.439
SII	2653.58 [1160.51, 4347.14]	2829.56 [1190.79, 4274.10]	1700.92 [833.47, 4398.94]	0.588
SOFA	6.00 [5.00, 9.00]	6.00 [5.00, 7.00]	10.00 [9.00, 11.00]	**<0.001**
APACHE_II	10.00 [8.00, 16.00]	10.00 [8.00, 12.00]	19.00 [15.00, 21.00]	**<0.001**
Clinical outcome				
28-day-mortality (%)	36 (40.4)	23 (33.8)	13 (61.9)	**0.04**

RA, rheumatoid arthritis; SAE, sepsis-associated encephalopathy; LOS, length of stay; CAD, coronary artery disease; WBC, white blood cell; CRP, C-reactive protein; NEU, neutrophil count; LYM, lymphocyte count; MONO, monocyte count; RBC, red blood cell; Hb, hemoglobin; PLT, platelet; ALB, albumin; GLB, globulin; PCT, procalcitonin; RF, rheumatoid factor; CK, creatine kinase; LDH, lactate dehydrogenase; Anti-CCP, anti-cyclic citrullinated peptide antibody; DAS28-CRP, Disease Activity Score in 28 joints using CRP; NAR, neutrophil-to-albumin ratio; NAR ×10, neutrophil-to-albumin ratio multiplied by 10 for analysis. NLR, neutrophil-to-lymphocyte ratio; PLR, platelet-to-lymphocyte ratio; SII, systemic immune-inflammation index; SOFA, Sequential Organ Failure Assessment; APACHE II, Acute Physiology and Chronic Health Evaluation II. Bold values indicate statistically significant differences (*p* < 0.05).

### 3.2 Machine learning-based feature selection for SAE prediction

**Figure 3:**
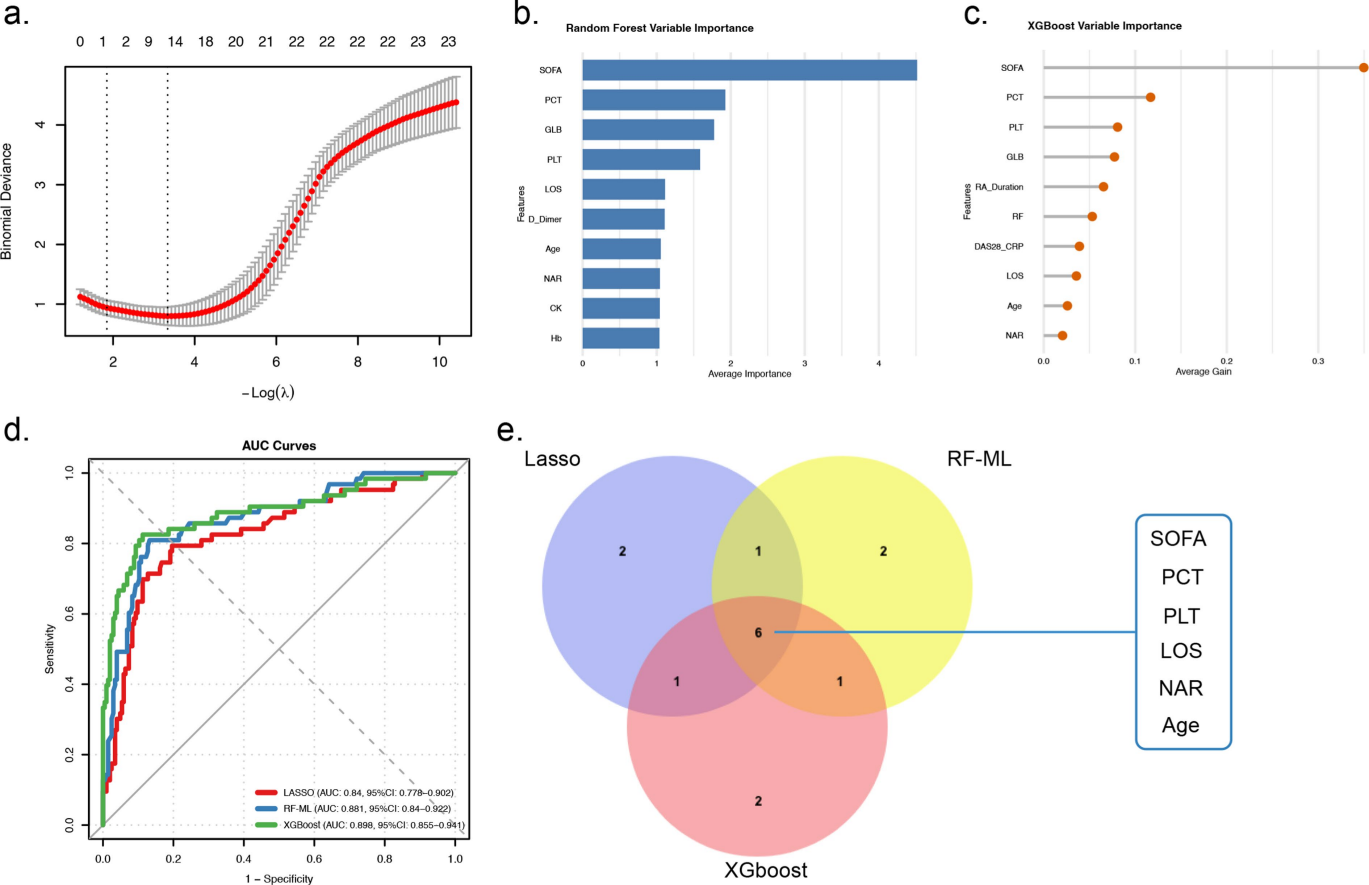
Feature selection and model evaluation based on machine learning. **(a)** LASSO cross-validation: Selection of the optimal penalty parameter *λ* using 5-fold cross-validation (repeated 3 times). **(b)** Random Forest importance: Variable ranking based on the Mean Decrease Gini. **(c)** XGBoost importance: Variable ranking based on the Average Gain. **(d)** AUC curves: Performance comparison among LASSO, RF-ML, and XGBoost models. **(e)** Venn diagram: Intersection of the three algorithms identifying six consensus predictors, including NAR. SAE, sepsis-associated encephalopathy; LASSO, least absolute shrinkage and selection operator; RF-ML, random forest machine learning; XGBoost, eXtreme Gradient Boosting; AUC, area under the ROC curve; NAR, neutrophil-to-albumin ratio; SOFA, Sequential Organ Failure Assessment; PCT, procalcitonin; PLT, platelet; LOS, length of stay.

Three machine learning algorithms, including LASSO regression, RF-ML, and XGBoost, were employed to identify potential predictors associated with SAE (Figures 3a-c). Model performance and stability were evaluated using repeated 5-fold cross-validation with three repetitions, and all models demonstrated good predictive performance, with AUC values ranging from 0.840 to 0.898 (Figure 3d). Feature importance analyses consistently identified systemic severity indicators, particularly SOFA score and PCT, as dominant predictors across the three algorithms. Notably, although NAR showed lower relative importance compared with SOFA, it was consistently retained by all three machine learning models, suggesting its potential robustness and clinical relevance in SAE prediction. To reduce algorithm-specific bias and improve feature stability, overlapping variables identified by all three algorithms were selected for subsequent analyses. As shown in the Venn diagram (Figure 3e), six shared features, including SOFA, PCT, PLT, LOS, NAR, and age, were ultimately identified as the final candidate predictors for downstream model construction.

### 3.3 Construction and validation of the integrated prediction model

**Figure 4:**
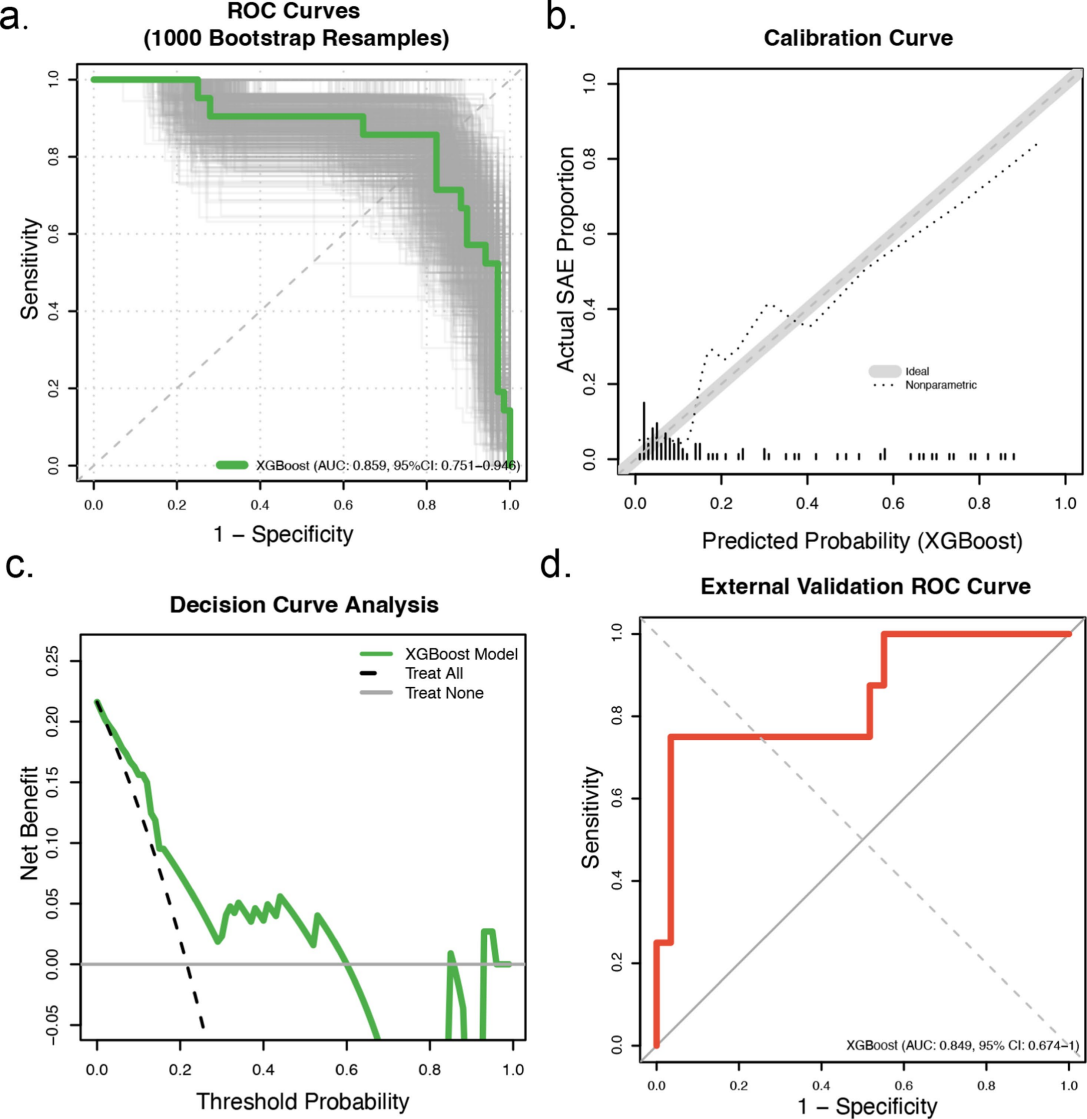
Performance evaluation of the XGBoost prediction model. **(a)** Receiver operating characteristic (ROC) curve of the XGBoost model with 1,000 bootstrap resamples. The green line represents the overall ROC curve (AUC: 0.859, 95% CI: 0.751–0.946), and the grey lines represent bootstrap resampling curves. **(b)** Calibration curve of the XGBoost model. **(c)** Decision curve analysis (DCA) of the XGBoost model. **(d)** ROC curve of the XGBoost model in the independent external validation cohort (AUC: 0.849, 95% CI: 0.674–1.000). SAE, sepsis-associated encephalopathy; ROC, receiver operating characteristic; AUC, area under the curve; CI, confidence interval; DCA, decision curve analysis; XGBoost, eXtreme Gradient Boosting.

Among the three machine learning algorithms evaluated, XGBoost demonstrated the highest predictive performance and was therefore selected to construct the final integrated prediction model using the six consensus variables (SOFA, PCT, PLT, LOS, NAR, and age). The final model achieved a bootstrap-corrected AUC of 0.859 (95% CI: 0.751–0.946). As shown in Figure 4a, the bootstrap resampling curves were closely distributed around the primary ROC curve. In addition, the calibration curve demonstrated good agreement between predicted and observed SAE risk (Figure 4b). DCA further indicated the potential clinical utility of the XGBoost model across a wide range of threshold probabilities (Figure 4c). To further evaluate the incremental predictive value of NAR, a baseline model excluding NAR was compared with the final model. The addition of NAR resulted in a modest improvement in discrimination (AUC 0.842 vs. 0.859), while significantly improving risk reclassification as demonstrated by the continuous NRI and IDI analyses (Supplementary Figure S2a and Supplementary Table S1).

A complete-case sensitivity analysis was subsequently performed after exclusion of patients with missing values in the final model variables. The resulting cohort comprised 87 patients. The XGBoost model yielded an AUC of 0.872 (95% CI: 0.775–0.970), consistent with the primary analysis based on the imputed dataset (Supplementary Figure S2b and Supplementary Table S2).

A conventional logistic regression model was additionally constructed using the same six predictors included in the final XGBoost model. The logistic regression model achieved an AUC of 0.849 (95% CI: 0.750–0.949), compared with 0.859 (95% CI: 0.759–0.960) for the XGBoost model. DeLong analysis demonstrated no significant difference between the two ROC curves (*p* = 0.773) (Supplementary Figure S2c). Nevertheless, the XGBoost model showed modest improvements in overall classification performance, particularly in specificity, accuracy, precision, and F1-score (Supplementary Table S3). Clinical utility analyses demonstrated a greater net benefit for the XGBoost model across most clinically relevant threshold probabilities (Supplementary Figure S2d). Reclassification analyses did not reveal significant improvements, with non-significant continuous NRI, and IDI values (Supplementary Table S4).

An independent external validation cohort from the Affiliated Hospital of Changchun University of Chinese Medicine was subsequently used for model validation. This cohort comprised 37 patients, including 8 SAE cases and 29 non-SAE cases. Baseline characteristics of the external validation cohort are summarized in Supplementary Table S5. The final XGBoost model maintained good discriminatory performance in the external cohort, achieving an AUC of 0.849 (95% CI: 0.674–1.000) (Figure 4d).

### 3.4 Interpretation of machine learning models using SHAP analyses

**Figure 5:**
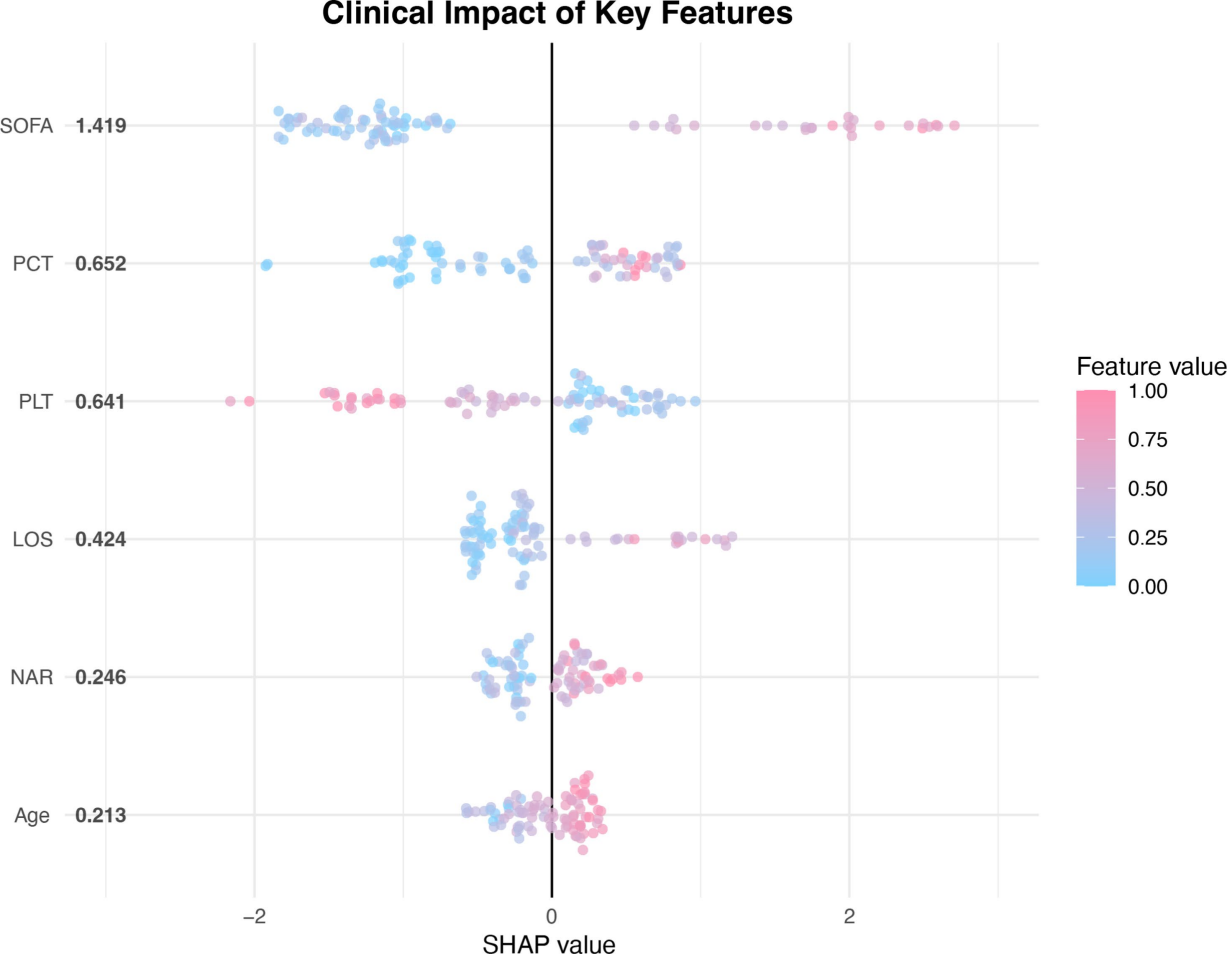
Model interpretation using SHAP summary plot. The SHAP summary plot illustrates the contribution of the six consensus variables to the XGBoost model’s output. Each dot represents an individual patient, with the color indicating the feature value (pink for high; blue for low). The horizontal position (SHAP value) indicates whether the feature is associated with an increased (positive) or decreased (negative) risk of SAE. SAE, sepsis-associated encephalopathy; SHAP, SHapley Additive exPlanations; NAR, neutrophil-to-albumin ratio; SOFA, Sequential Organ Failure Assessment; PCT, procalcitonin; PLT, platelet count.

SHAP analysis was performed to interpret the final XGBoost model and quantify the contribution of each feature to SAE prediction (Figure 5). The SHAP summary plot demonstrated that higher SOFA, PCT, LOS, age, and NAR values were associated with an increased predicted risk of SAE, whereas higher PLT levels were associated with a reduced risk. Among all variables, SOFA and PCT exhibited the greatest influence on model output. Although the contribution of NAR was relatively smaller compared with SOFA and PCT, elevated NAR levels consistently showed positive SHAP values, indicating a stable association with increased SAE risk within the XGBoost model.

### 3.5 Development of a web-based clinical prediction tool

**Figure 6:**
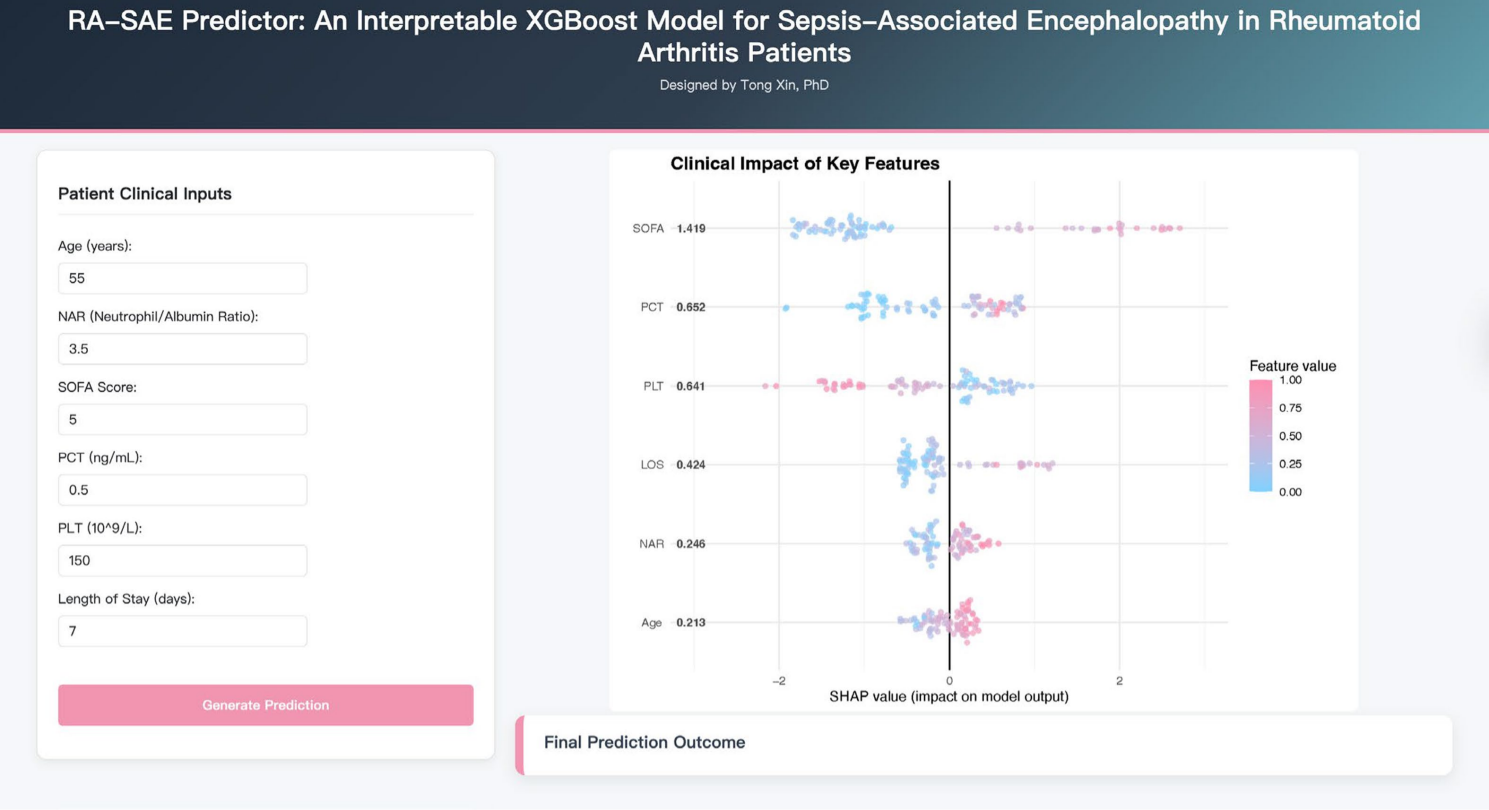
User interface of the interactive web-based dynamic calculator for predicting SAE risk. SAE, sepsis-associated encephalopathy.

To facilitate clinical application of the final XGBoost model, a web-based dynamic prediction tool was developed (Figure 6). The interactive application is publicly available at RA-SAE Risk Calculator^1^. By entering six routinely available clinical variables (Age, NAR, SOFA, PCT, PLT, and LOS), clinicians can obtain individualized risk predictions for SAE in real time. This web-based tool provides an intuitive interface for implementing the XGBoost model in clinical practice and may assist early risk stratification and clinical decision-making in patients with sepsis.

### 3.6 Correlation analysis and 28-day survival analysis

**Figure 7:**
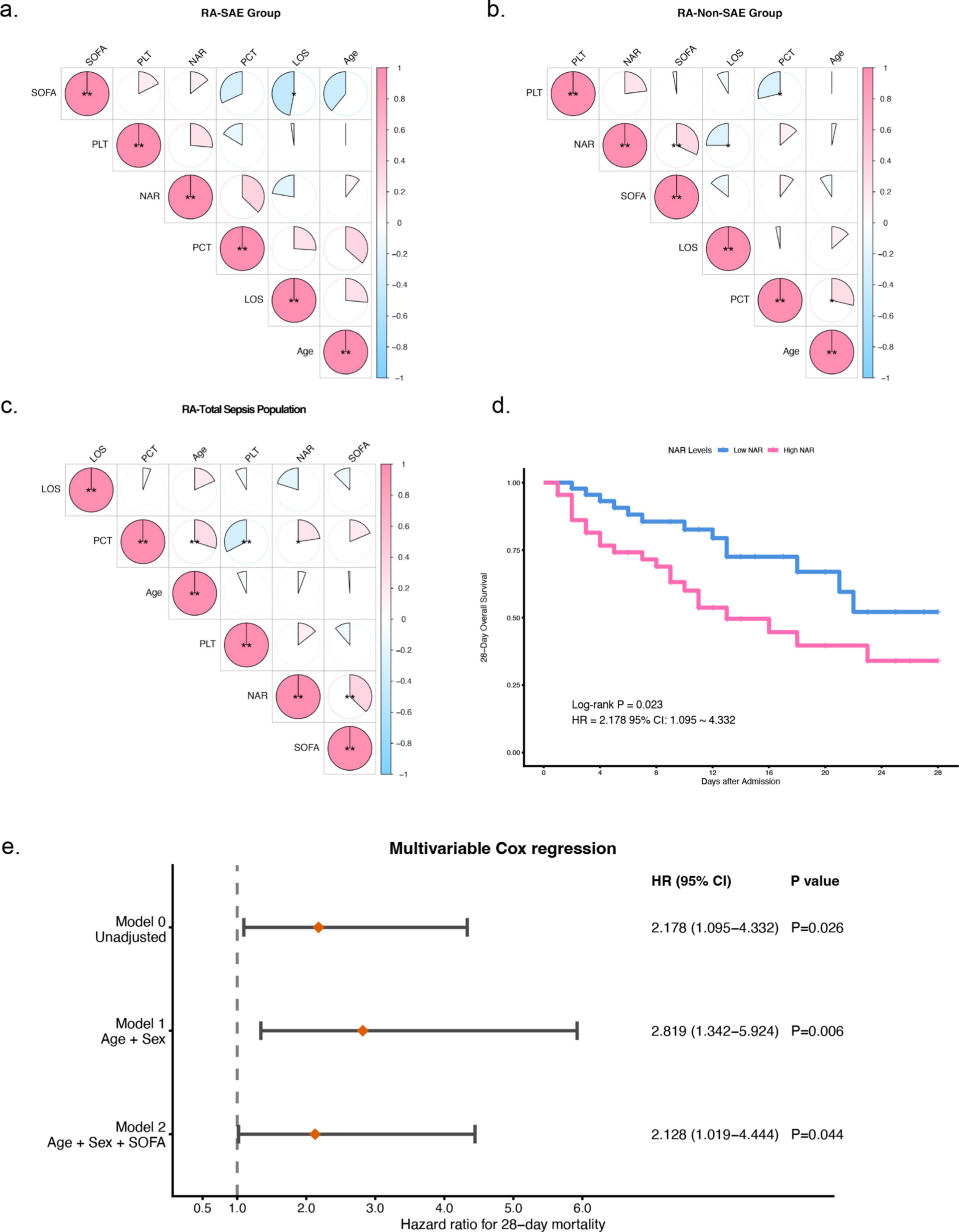
Correlation matrices of core clinical variables across different patient cohorts. Spearman correlation matrices are displayed for **(a)** the RA–SAE group, **(b)** the RA-non-SAE group, and **(c)** the RA–total sepsis population, with the filled area and color intensity of the pies reflecting the magnitude and direction of the correlation coefficients. **(d)** Kaplan–Meier survival curves indicating the cumulative 28-day overall survival probability stratified by the median baseline NAR cutoff value (Low NAR in blue vs. High NAR in pink). **(e)** Forest plot of unadjusted and multivariable Cox proportional hazards regression analyses evaluating the association between median-based NAR grouping and 28-day mortality. Patients were categorized into high- and low-NAR groups according to the median baseline NAR value. Model 1 was adjusted for age and sex, whereas Model 2 was additionally adjusted for SOFA score based on Model 1. Hazard ratios (HRs) and 95% confidence intervals (CIs) are shown. RA, rheumatoid arthritis; SAE, sepsis-associated encephalopathy; SOFA, Sequential Organ Failure Assessment; PLT, platelet count; NAR, neutrophil-to-albumin ratio; PCT, procalcitonin; LOS, length of stay; HR, Hazard ratios; Cis, confidence intervals.

Spearman correlation analysis revealed distinct correlation patterns among clinical variables across the SAE, non-SAE, and overall sepsis cohorts (Figures 7a–c). In the non-SAE group and the overall cohort, consistent correlation patterns were observed, including a negative correlation between PLT and PCT, a positive correlation between PCT and age, and a positive correlation between NAR and SOFA score (Figures 7b,c). In addition, NAR was negatively correlated with LOS in the non-SAE group and positively correlated with PCT in the overall cohort. In contrast, these correlation patterns were substantially attenuated in the SAE group, where only a significant negative association between SOFA score and LOS was observed (Figure 7a). This finding may be partly attributable to the relatively small sample size and higher early mortality rate in the SAE cohort.

The prognostic value of NAR was further evaluated using survival analyses (Figure 7d). Kaplan–Meier analysis demonstrated that patients in the high NAR group had significantly lower 28-day survival compared with those in the low NAR group (*p* = 0.023, log-rank test), with an early separation of survival curves. Univariate Cox regression analysis further confirmed that elevated baseline NAR was associated with increased risk of 28-day mortality (HR = 2.178, 95% CI: 1.095–4.332). Additional multivariable Cox regression analyses were subsequently performed (Figure 7e). High NAR was associated with a higher risk of 28-day mortality after adjustment for age and sex (HR = 2.819, 95% CI: 1.342–5.924). This association remained significant after further adjustment for SOFA score (HR = 2.128, 95% CI: 1.019–4.444). These findings suggest that NAR may serve as a useful risk stratification marker for short-term mortality in patients with RA and sepsis.

## 4 Discussion

To our knowledge, this is the first real-world study specifically investigating SAE in patients with RA. In the retrospective cohort, we identified distinct clinical characteristics associated with SAE development and established an XGBoost-based predictive model with robust discriminative performance. Among the identified predictors, the NAR emerged as a clinically accessible indicator associated with both SAE occurrence and increased 28-day mortality. These findings provide preliminary evidence that RA patients with sepsis may represent a unique high-risk subgroup for SAE and support the potential utility of machine learning-assisted risk stratification in this vulnerable population.

In this study, SAE occurred in 23.6% of RA patients with sepsis, and was associated with a 28-day mortality of 61.9%, nearly twice that observed in non-SAE patients and substantially higher than the approximately 40% mortality reported in previous SAE cohorts (5, 23). This disproportionate mortality burden suggests that SAE in RA patients is not merely an isolated neurological manifestation, but rather a sentinel of profound systemic deterioration and impending multi-organ failure. Several interrelated factors may contribute to this exceptional vulnerability. Chronic immune dysregulation and persistent inflammatory burden in RA progressively impair physiological reserve long before sepsis onset, while advanced age, multimorbidity, and prolonged exposure to glucocorticoids or biologic DMARDs further compromise host defense and organ resilience (24, 25). Consequently, once severe infection develops, RA patients may be less capable of tolerating the overwhelming inflammatory, endothelial, and microcirculatory disturbances associated with sepsis, thereby accelerating progression toward brain dysfunction and adverse outcomes (26, 27). Importantly, these findings also highlight a critical yet often underrecognized clinical challenge in frontline and resource-limited settings, where RA patients with sepsis are frequently first evaluated. In the absence of specialist neurocritical care or rheumatology support, nonspecific manifestations such as delirium, fluctuating consciousness, or subtle cognitive decline may be easily misattributed to metabolic encephalopathy, sedative exposure, or underlying chronic disease, potentially leading to delayed recognition of SAE and missed opportunities for timely intervention. Therefore, the development of simple, rapidly obtainable, and clinically interpretable prognostic tools, such as the NAR-integrated model proposed in this study, may be particularly valuable for facilitating early risk stratification, neurological monitoring, and escalation of care before irreversible deterioration occurs.

The biological relevance of NAR in RA patients with SAE likely reflects the chronic inflammatory state of RA and its amplification during sepsis. Neutrophils play a central role in RA pathogenesis and exhibit reactive oxygen species production, proinflammatory cytokine release, delayed apoptosis, and increased neutrophil extracellular traps (NETs) formation, thereby contributing to persistent synovial inflammation and tissue destruction (28–30). Hypoalbuminemia is also common in RA and may reflect the chronic inflammation and reduced physiological reserve (5, 31, 32). Therefore, NAR integrates neutrophil-driven inflammatory and reduced physiological reserve associated with low albumin levels, potentially providing a broader reflection of disease severity than isolated inflammatory markers (5). This concept is consistent with the growing recognition that integrating traditional clinical indicators with emerging biomarkers may improve risk stratification in complex diseases and facilitate more individualized prognostic assessment (33). During sepsis, this pre-existing inflammatory imbalance may be further amplified. Excessive systemic inflammation, oxidative stress, endothelial dysfunction, and microcirculatory disturbances may contribute to blood–brain barrier disruption and neuroinflammatory responses, thereby increasing susceptibility to SAE (34, 35). These vascular alterations may represent an important mechanism linking systemic inflammation to neurological injury (34–37). Neutrophil activation and NET-associated endothelial injury may promote microvascular dysfunction, whereas hypoalbuminemia may aggravate vascular leakage, cerebral edema, and impaired antioxidant defense (35). Thus, elevated NAR may reflect the combined burden of immune dysregulation and physiological vulnerability in RA patients at risk of SAE.

Treatment-related factors may also influence this association. Glucocorticoids and DMARDs can affect immune function, infection susceptibility, and systemic inflammatory responses, thereby potentially influencing both NAR levels and SAE risk (3, 24, 25). In our cohort, baseline glucocorticoid use was recorded only as a binary variable, whereas glucocorticoid dose and cumulative exposure were unavailable. DMARD-related variables were not included in the final analysis because of substantial missingness across participating centers. Therefore, residual confounding related to antirheumatic therapy cannot be completely excluded, and potential effect modification by glucocorticoid dose or DMARD type cannot be assessed.

Moreover, elevated NAR was significantly associated with poorer short-term outcomes in our cohort. Kaplan–Meier analysis demonstrated lower 28-day survival among patients with high NAR levels, while Cox regression further showed that elevated baseline NAR was associated with increased 28-day mortality. These findings further support the prognostic relevance of NAR in RA patients with SAE. To our knowledge, this is the first study to evaluate the clinical significance of NAR in SAE among patients with RA, extending the applicability of this readily accessible biomarker to a novel high-risk population.

In the present study, machine learning approaches demonstrated favorable performance for predicting SAE in patients with RA and sepsis, with the XGBoost model showing the best overall discriminative ability and calibration performance. Compared with traditional regression-based methods, machine learning algorithms are better suited to capture complex nonlinear relationships and interactions among clinical variables, which is particularly important in critically ill patients with multifactorial pathophysiological disturbances. Given that SAE is driven by interconnected processes involving systemic inflammation, immune dysregulation, endothelial dysfunction, metabolic abnormalities, and organ failure, conventional linear models may be insufficient for precise risk stratification. Importantly, our model was constructed using routinely available clinical variables, enhancing its potential applicability in real-world critical care settings. Bootstrap resampling confirmed model stability, while DCA demonstrated favorable clinical utility across a broad range of threshold probabilities. In addition, SHAP analysis improved model interpretability by quantifying the contribution of individual predictors to SAE risk. Among these variables, SOFA, PCT, LOS, age, and NAR were positively associated with predicted SAE risk, whereas higher PLT levels appeared protective. Notably, NAR emerged as an important contributor, further supporting its potential role as an accessible biomarker integrating inflammatory activation and physiological vulnerability. Furthermore, the development of a web-based dynamic prediction tool may facilitate real-time bedside risk assessment, thereby enabling earlier neurological monitoring, individualized therapeutic strategies, and optimized allocation of critical care resources (38). More broadly, recent advances in digital health have highlighted the potential of predictive algorithms to support clinical decision-making in rheumatology; however, successful implementation will require careful consideration of model interpretability, workflow integration, clinician acceptance, and ongoing external validation before widespread adoption in routine practice (38, 39). Nevertheless, external validation and prospective multicenter studies remain necessary before widespread clinical implementation.

Correlation analyses demonstrated distinct patterns between SAE and non-SAE groups. In the non-SAE group and the overall cohort, relatively consistent associations were observed among key clinical variables, including negative correlations between PLT and PCT, and positive correlations between PCT and age, as well as between NAR and SOFA score. In contrast, these correlation structures appeared attenuated in the SAE group, where only a significant association between SOFA score and LOS was retained. This reduction in correlation complexity may partly reflect the severe physiological instability in SAE patients, as well as the influence of early mortality leading to incomplete manifestation of stable inter-variable relationships. Within the overall cohort, NAR showed consistent associations with established markers of disease severity. Higher NAR values were positively correlated with SOFA score and PCT, while negatively correlated with PLT, suggesting that NAR reflects both inflammatory burden and relative physiological reserve. These findings are biologically plausible, as neutrophil activation is central to systemic inflammatory responses, whereas hypoalbuminemia is associated with impaired antioxidant capacity and increased vascular permeability (31, 34). Collectively, these results support the internal consistency of NAR as an integrated marker of immune activation and host response and provide further statistical support for its inclusion in predictive modeling of SAE in RA patients with sepsis.

Several limitations of this study should be acknowledged. First, SAE diagnosis was based on retrospective clinical records, and standardized delirium assessment tools such as the Confusion Assessment Method for the Intensive Care Unit (CAM-ICU) were not routinely available. This may have increased the risk of diagnostic misclassification, especially in patients with RA, in whom autoimmune or inflammatory neurological manifestations may resemble SAE. Although cases were reviewed by senior rheumatologists and neurocritical care specialists with strict exclusion of alternative neurological causes, residual diagnostic uncertainty cannot be completely excluded. Second, the relatively small sample size and limited number of SAE events may have affected the statistical stability of the model. Although repeated cross-validation, bootstrap resampling, and external validation were performed, validation in larger multicenter cohorts remains necessary. In addition, the limited number of outcome events restricted the number of covariates that could be reliably incorporated into multivariable survival analyses, and therefore residual confounding cannot be completely excluded. Third, the study population was exclusively derived from a Chinese cohort. Given potential differences in genetic background, disease phenotypes, and clinical management strategies, the generalizability of our findings to other ethnic populations may be limited. Fourth, the study period spanned the COVID-19 pandemic (2020–2025), which may have influenced case mix, infection etiology, ICU management practices, and clinical outcomes in ways that are not fully quantifiable, thereby introducing additional clinical heterogeneity that could not be completely accounted for in this retrospective study. Fifth, only baseline NAR measured within the first 24 h after sepsis onset was included in the present analysis. Given the highly dynamic nature of sepsis, characterized by rapidly evolving inflammatory responses and organ dysfunction, a single baseline measurement may not fully capture the temporal trajectory of disease progression. Because follow-up measurements were obtained at non-standardized time points and may have been influenced by subsequent therapeutic interventions, reliable analysis of NAR trajectories at predefined time points was not feasible. Therefore, future studies should investigate whether dynamic changes in NAR during the first 48–72 h after ICU admission, compared with baseline measurements alone, provide additional prognostic value for SAE risk stratification.

## 5 Conclusion

In conclusion, this study developed, internally validated, and externally evaluated a machine learning-based predictive model for SAE in RA patients with sepsis, identifying six consensus predictors including the novel composite index NAR. The XGBoost model demonstrated robust discriminative performance and was translated into a web-based clinical tool to facilitate bedside risk stratification. Our findings support the integration of NAR into routine prognostic assessment of RA patients with sepsis, potentially enabling earlier recognition of neurological complications and more individualized management approaches. Future prospective multicenter studies are warranted to confirm its robustness and generalizability in larger and more diverse populations and further elucidate the mechanistic links between neutrophil activation, nutritional status, and sepsis-associated brain dysfunction in autoimmune populations.

## Data Availability

The original contributions presented in the study are included in the article/Supplementary material, further inquiries can be directed to the corresponding author/s.

## Footnotes

1
https://dr-xintong.shinyapps.io/RA_SAE_risk/
