# Supplementary material for: Neutrophil-to-albumin ratio as a predictor of sepsis-associated encephalopathy in patients with rheumatoid arthritis: an exploratory machine learning study
Source: Front Neurol. 2026 Jul 23;17:1884282. doi: 10.3389/fneur.2026.1884282 (PMC13441812; doi:10.3389/fneur.2026.1884282)
Supplement: Supplementary file 1 [file Table_1.DOCX]

Supplementary Material

# 1 Supplementary Figure

**Supplementary Figure S1.** Data Quality Control and Pre-processing. (a) Missingness Pattern: Visualization of initial missing data distribution across all variables. (b) Parameter Optimization: Selection of the optimal k value for the imputation model based on the minimum Root Mean Square Error (RMSE). (c) Imputation Fidelity: Boxplots comparing the distributions of key variables (Anti-CCP, D-Dimer, PCT, and RF) before and after hybrid kNN-Random Forest imputation. (d) Imputed Value Distribution: Matrix plots showing the spatial integration of imputed values (red) within the observed data (blue). (e) Outlier Mitigation: Comparison of continuous variables before and after Winsorization to limit the impact of extreme values.

**Supplementary Figure S2.** Incremental Predictive Value of NAR, Sensitivity Analyses, and Model Performance Comparisons. (a) Receiver operating characteristic (ROC) curves comparing the baseline model and the NAR-enhanced model. The baseline model incorporated SOFA score, PCT, PLT, LOS, and age, whereas the NAR-enhanced model additionally included NAR. (b) ROC curve of the complete-case sensitivity analysis. Patients with missing values in any of the six variables included in the final XGBoost model were excluded, resulting in a complete-case cohort of 87 patients. (c) ROC curve comparison between the XGBoost model and a conventional logistic regression model. Model discrimination was assessed using the area under the ROC curve (AUC), and 95% confidence intervals were calculated using the DeLong method. (d) Decision curve analysis (DCA) comparing the clinical utility of the XGBoost and logistic regression models across a range of threshold probabilities. Note: For panel (b), the AUC and its 95% confidence interval were estimated using 1,000 bootstrap resamples. For panels (a) and (c), AUC confidence intervals were estimated using the DeLong method, and ROC curves were compared using DeLong’s test. Abbreviations: AUC, area under the receiver operating characteristic curve; DCA, decision curve analysis; LOS, length of stay; NAR, neutrophil-to-albumin ratio; PCT, procalcitonin; PLT, platelet count; ROC, receiver operating characteristic; SOFA, Sequential Organ Failure Assessment; XGBoost, eXtreme Gradient Boosting.

# 2 Supplementary Table

**Supplementary Table S1. Incremental predictive value of NAR for the XGBoost model.**

| Metric | Estimate | CI_95 | *p*-value |
| --- | --- | --- | --- |
| Continuous NRI | 1.2 | 0.796 – 1.605 | **<0.001** |
| IDI | 0.058 | 0.031 – 0.086 | **<0.001** |

Note: NRI, Net Reclassification Improvement; IDI, Integrated Discrimination Improvement. Incremental predictive performance was evaluated by comparing the baseline model (SOFA, PCT, PLT, LOS, and age) with the NAR-enhanced model (baseline model plus NAR).

**Supplementary Table S2. Sensitivity analysis of the final XGBoost model.**

| Analysis | Sample size (n) | AUC | 95% CI |
| --- | --- | --- | --- |
| Primary analysis (imputed dataset) | 89 | 0.859 | 0.751-0.946 |
| Complete-case sensitivity analysis | 87 | 0.872 | 0.775-0.970 |

Note: The primary analysis was performed using the imputed dataset (n = 89). To evaluate the impact of missing-data imputation, a complete-case sensitivity analysis was conducted by excluding patients with missing values in the six variables included in the final XGBoost model (age, LOS, NAR, SOFA score, PCT, and PLT). Only two patients were excluded, yielding a complete-case cohort of 87 patients. The model was subsequently rebuilt and evaluated using the same analytical procedures. AUC, area under the receiver operating characteristic curve; CI, confidence interval.

**Supplementary Table S3. Performance comparison between the XGBoost and logistic regression models.**

| Metric | XGBoost | Logistic_Regression |
| --- | --- | --- |
| AUC | 0.859 | 0.849 |
| Sensitivity | 0.857 | 0.952 |
| Specificity | 0.824 | 0.706 |
| Accuracy | 0.831 | 0.764 |
| Precision | 0.6 | 0.5 |
| NPV | 0.949 | 0.98 |
| F1-score | 0.706 | 0.656 |

Note: AUC indicates the area under the receiver operating characteristic curve; PPV, positive predictive value; NPV, negative predictive value. Sensitivity, specificity, accuracy, precision, NPV, and F1-score were calculated using the optimal probability threshold determined for each model.

**Supplementary Table S4. Net reclassification improvement (NRI) and integrated discrimination improvement (IDI) analyses comparing the XGBoost and logistic regression models.**

| Metric | Estimate | CI_95 | *p*-value |
| --- | --- | --- | --- |
| Continuous NRI | -0.430 | -0.910 – 0.050 | 0.079 |
| IDI | 0.003 | -0.108 – 0.114 | 0.952 |

Note: AUC indicates the area under the receiver operating characteristic curve; PPV, positive predictive value; NPV, negative predictive value. Sensitivity, specificity, accuracy, precision (PPV), NPV, and F1-score were calculated using the optimal probability threshold determined for each model.

**Supplementary Table S5. Baseline characteristics of the external validation cohort.**

|  | Overall (n=37) | Non-SAE (n=29) | SAE (n=8) | *p*-value |
| --- | --- | --- | --- | --- |
| Age, years | 66.00 [59.00, 75.00] | 62.00 [59.00, 75.00] | 68.50 [64.25, 74.50] | 0.567 |
| LOS, days | 9.00 [8.00, 15.00] | 10.00 [8.00, 15.00] | 8.00 [6.50, 11.50] | 0.238 |
| NEU (×10^9^/L) | 12.94 [8.20, 16.30] | 12.94 [8.20, 16.00] | 13.66 [9.24, 16.59] | 0.768 |
| ALB (g/L) | 29.90 [25.40, 32.40] | 31.00 [27.00, 32.60] | 25.40 [24.60, 28.00] | **0.029** |
| PCT (ng/mL) | 2.13 [1.12, 3.76] | 1.46 [1.05, 2.90] | 2.74 [2.19, 3.91] | 0.101 |
| PLT (×10^9^/L) | 169.00 [96.00, 207.00] | 176.00 [96.00, 238.00] | 135.00 [91.00, 160.25] | 0.216 |
| NAR (×10) | 4.63 [2.65, 5.89] | 4.36 [2.64, 5.77] | 5.65 [3.14, 6.71] | 0.238 |
| SOFA | 9.00 [6.00, 10.00] | 8.00 [6.00, 10.00] | 9.50 [9.00, 12.00] | **0.025** |

Note: SAE, sepsis-associated encephalopathy; LOS, length of stay; NAR, neutrophil-to-albumin ratio; NAR ×10, neutrophil-to-albumin ratio multiplied by 10 for analysis; SOFA, Sequential Organ Failure Assessment; PCT, procalcitonin; PLT, platelet count.
